# WHAT DETERMINES THE USE OF MEANING FOCUSED COPING IN DEMENTIA CAREGIVING?

**DOI:** 10.1093/geroni/igz038.2981

**Published:** 2019-11-08

**Authors:** Doris Yu, Sheung Tak cheng

**Affiliations:** The Education University of Hong Kong, Hong Kong, Hong Kong

## Abstract

Meaning-focused coping has been identified as an important factor contributing to more positive adjustment and health outcomes for family caregivers of persons with dementia. Yet, there is less evidence about what determines the use of this coping strategy. Based on the Meaning Making Model, this quantitative exploratory study identified the relationship between meaning focused coping and intrinsic motivation towards caregiving, quality of relationship between the care dyads and level of religiosity. Two hundreds and five family caregivers of PWD were recruited from a geriatric clinic from March 2018 – Feb 2019. A battery of questionnaires including the Meaning-Focused Coping Scale, Relationship Quality Scale, the Duke University Religion Index and Intrinsic Motivations to Care was administered in face-to-face interview. By using hierarchical regression analysis to control the effects of caregivers’ demographic profile, caregiving history, clinical severity of dementia, and level of neuro-psychiatric symptoms, the results indicated that a higher intrinsic motivation to caregiving (β = 1.457, p = 0.044), better dyadic relationship quality (β = 0.768, p = 0.004) and a higher level of religiosity (β = 0.969, p = 0.001) are independently related to a higher likelihood of using meaning-focused coping. The findings shift the paradigm of caregiver support from a deficit model to helping them to appraise the meaning of caregiving in dyadic relational, spiritual and motivational perspectives. The application of strength-based and person-centered approach to caregiver supportive program will be discussed.

